# Gender-Related Differences in the Hospitalization Outcomes for Gastroparesis

**DOI:** 10.7759/cureus.86239

**Published:** 2025-06-17

**Authors:** Ahmed Ali Aziz, Rehan Shah, Muhammad Ali Aziz, Muhammad Amir, Ijlal Akbar Ali

**Affiliations:** 1 Internal Medicine, INTEGRIS Health Baptist Medical Center, Oklahoma City, USA; 2 Internal Medicine - Rheumatology, Bayonne Medical Center, Bayonne, USA; 3 Internal Medicine, University of Kentucky College of Medicine, Lexington, USA; 4 Gastroenterology, INTEGRIS Health Baptist Medical Center, Oklahoma City, USA; 5 Digestive Diseases and Nutrition, University of Oklahoma Health Sciences Center, Oklahoma City, USA

**Keywords:** acute sepsis, gastroparesis, gender-related differences, hospital outcomes, icu admission, length of hospital stay, national inpatient sample (nis), shock, venous thromboembolism (vte)

## Abstract

Background

Gastroparesis (GP) is a chronic condition in which the stomach takes too long to empty its contents into the small intestine, despite the absence of any physical blockage. Females are generally more susceptible to developing GP than males. The objective of this study was to compare clinical outcomes between male and female patients hospitalized with GP using the Nationwide Inpatient Sample (NIS) database.

Methods

Using the NIS databases from 2020 to 2022 and the International Classification of Diseases, Tenth Edition Revision (ICD-10) codes, we performed a retrospective study of adult patients admitted with GP. We compared inpatient outcomes of GP between males and females. All-cause in-hospital mortality was the primary outcome. Secondary outcomes were total hospitalization cost adjusted to the year 2022, length of stay (LOS), incidence of sepsis, acute renal failure (ARF), shock, and need for intensive care unit (ICU) admission. We used STATA, version 16.1 (StataCorp LLC, College Station, TX), to perform the statistical analyses. Multivariate logistic regression analysis was conducted to assess whether gender was an independent predictor for these outcomes and to adjust for any confounders.

Results

It was noted that 31,114 adult patients were admitted for GP from 2020 to 2022; 23,886 (76.77%) were females, and 7,228 (23.23%) were males. The mean age of both males and females was 47.2 years. Males had a higher prevalence of diabetes mellitus type 1 (DM1), diabetes mellitus type 2 (DM2), congestive heart failure (CHF), chronic kidney disease (CKD), and smoking/tobacco use. Females had a higher prevalence of prior cerebrovascular accident (CVA) and obesity. We found that female patients with GP had significantly longer LOS (+0.88 days, 95% CI: 0.53 - 1.29, P <0.01), higher total hospitalization costs (+$9,129.4, 95% CI: 4,946.0-13,312.7, P <0.01), and higher likelihood of venous thromboembolism (VTE) (adjusted odds ratio (aOR) 1.69, 95% CI: 0.83-3.44, P=0.147) as compared to males. Female patients had lower odds of developing sepsis (aOR: 0.60, 95% CI: 0.43-0.85, P <0.01), ARF (aOR 0.48, 95% CI: 0.41-0.56, P <0.01), shock (aOR: 0.54, 95% CI: 0.24-1.22, P=0.143), ICU admission (aOR 0.73, 95% CI: 0.57-0.92, P <0.01), and in-hospital mortality (aOR: 0.15, 95% CI: 0.05-0.45, P <0.01) as compared to males.

Conclusions

We found that female patients had longer hospital LOS, total hospitalization charges, and a higher risk of VTE, while males had a higher risk of ARF, sepsis, shock, ICU admission, and all-cause in-hospital mortality. Although females are more frequently hospitalized for GP, males had significantly poorer clinical outcomes as compared to females. Our findings indicate that male patients with GP experience worse inpatient outcomes and require more aggressive treatment to reduce the risk of mortality and morbidity.

## Introduction

Gastroparesis (GP) is characterized by the delayed emptying of solid food from the stomach in the absence of mechanical obstruction [1]. This condition has become increasingly common in recent years, with the prevalence rising to 267.7 per 100,000 United States (U.S.) adults in 2018 [2,3]. Symptoms of GP include nausea, vomiting, bloating, early satiety, postprandial fullness, and upper abdominal pain [4]. The pathophysiology of GP remains unclear and is still under investigation [5]. Treatment of GP involves dietary modifications such as eating small portions of food more frequently and using medications such as prokinetic agents like metoclopramide [5]. The three most common etiologies of GP include diabetes mellitus (DM), history of gastric surgery, and idiopathic GP [2, 6].

Over the years, the incidence and prevalence of GP have been increasing, leading to increased hospitalizations of patients with GP. This creates a need to understand this disease and its inpatient hospitalization outcomes better. There are limited studies that study epidemiologic characteristics and outcomes of GP in males and females hospitalized with GP. Hence, we conducted a study using the Nationwide Inpatient Sample (NIS) database to determine gender-specific outcomes in adult patients hospitalized with GP.

## Materials and methods

Data source

We conducted a retrospective analysis using data from the NIS covering the years 2020 to 2022. The NIS is the largest publicly available inpatient healthcare database in the U.S., maintained by the Healthcare Cost and Utilization Project (HCUP). It includes data from 48 states and represents over 98% of the U.S. population. Approximately seven million hospitalizations are recorded in the NIS each year, drawn from the State Inpatient Databases. The NIS is a 20% stratified sample of all U.S. hospital admissions, allowing for reliable national estimates of comorbidities and disease prevalence. Each discharge is weighted to represent the national population, with the weighting based on the ratio of total discharges from all acute care hospitals to the number in the 20% sample. When weighted, the NIS reflects around 35 million hospitalizations annually across the country [7]. The dataset includes detailed information such as patient demographics, including gender, admission characteristics, length of stay (LOS), discharge diagnoses, and hospital charges. For this study, we used the International Classification of Diseases, Tenth Revision (ICD-10) codes from the World Health Organization (WHO) for diagnostic identification [8].

Study population

The ICD-10-CM code ‘K31.84’ was used to identify all patients with a primary discharge diagnosis of GP. We then used the ICD-10 code ‘R11’ to identify all patients with a primary discharge diagnosis of nausea and/or vomiting, but these patients also had a secondary diagnosis of GP [8]. This way we inferred that in these patients the severe nausea and vomiting were secondary to the exacerbation of underlying GP. We combined these patients into a single set of patient population that comprised our study population. We divided our study population by biological sex into males and females. Patients were excluded if they were transferred from another hospital, had an elective admission, were <18 years old, or died on the day of admission.

Study variables

Our study variables included age, primary payer (Medicare, Medicaid, private, self-pay), income quartile, hospital location, biologic sex, hospital bed size, hospital teaching status, and race (White, Black, Hispanic, Asian/Pacific Islander, Native American, Other). The NIS classifies any race that is not White, Black, Hispanic, Asian/Pacific Islander, or Native American as "Other." We used the Charlson Comorbidity Index (CCI) to assess the burden of comorbidities.

Study outcomes

All-cause in-hospital mortality was the primary outcome. Secondary outcomes included mean LOS, mean total hospitalization charges adjusted to year 2022, need for intensive care unit (ICU) admission, incidence of acute renal failure (ARF), sepsis, acute venous thromboembolism (VTE), and shock.

Statistical analysis

Statistical analyses were conducted using STATA version 16.1 (StataCorp, College Station, Texas, USA), which enables the use of NIS data to generate nationally representative findings. Continuous variables were analyzed using the Student’s t-test, while categorical variables were compared using the chi-square test. Univariate analysis was performed to explore the association between individual variables and the outcomes. Variables with a p-value less than 0.2 were included in the multivariate regression model. This model was then used to estimate outcomes while adjusting for potential confounders, including the CCI, age, race, hospital bed size, hospital teaching status, hospital location, and insurance status. Logistic regression was applied for binary outcomes, and linear regression was used for continuous outcomes. A p-value of less than 0.05 was considered statistically significant across all analyses.

## Results

Patient and hospital characteristics

**Figure 1:**
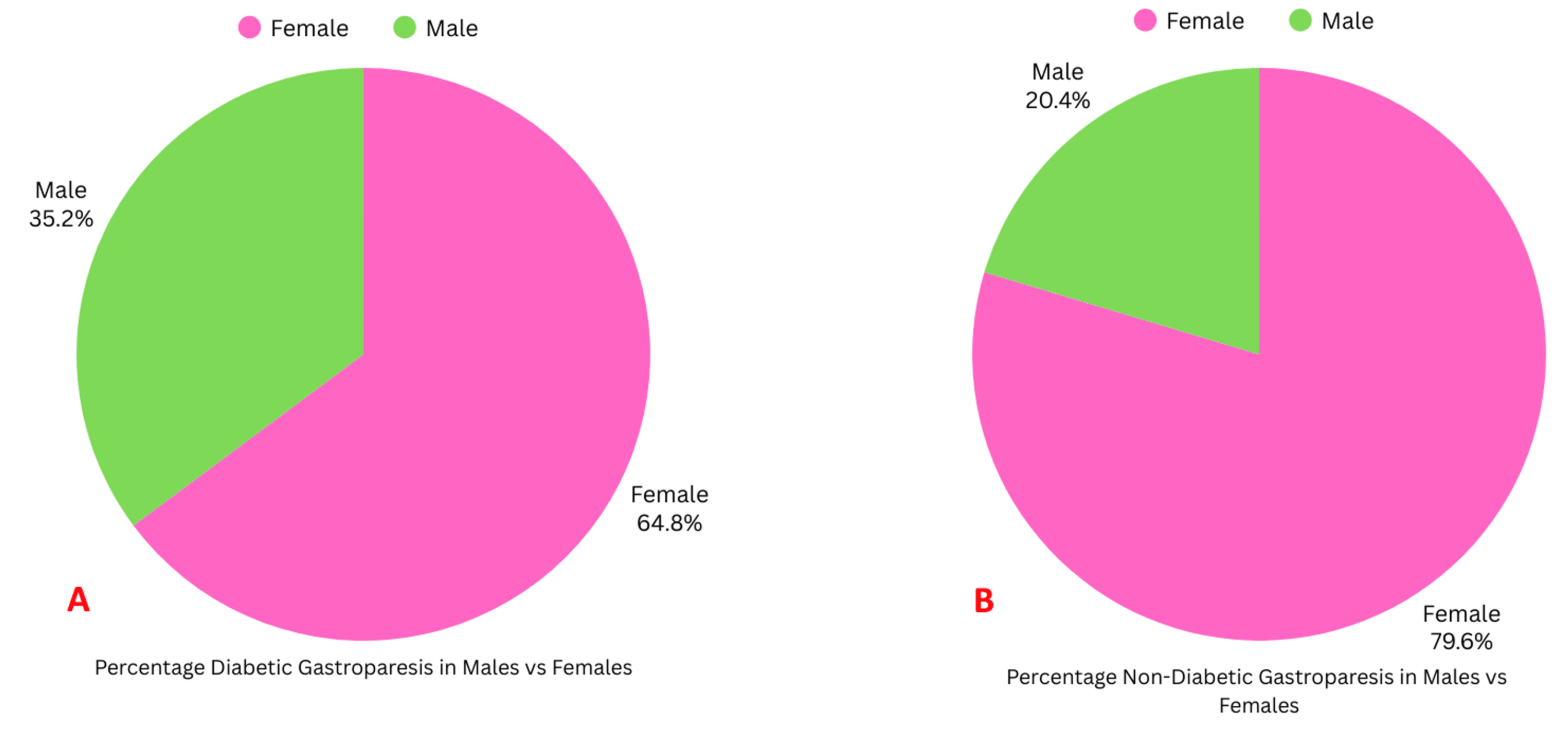
Percentage-wise distribution of males and females with diabetic and nondiabetic gastroparesis A: Percentage of diabetic gastroparesis in males vs. females; B: Percentage of non-diabetic gastroparesis in males vs. females

The 2020-2022 NIS databases had over 98 million hospital-weighted discharges, of which 31,114 patients had a principal discharge diagnosis of GP or nausea and/or vomiting with a secondary diagnosis of GP; 23,886 (76.77%) patients were females, and 7,228 (23.23%) patients were males. The mean age of both males and females with GP was the same: 47.2 years; 5,995 (19.3%) patients had diabetic GP, while 25,119 (80.7%) patients had non-diabetic GP. Among patients with diabetic GP, 3,885 (64.80%) were females and 2,110 (35.20%) were males. Among patients with non-diabetic GP, 20,002 (79.63%) were females and 5,117 (20.37%) were males (Figures 1A, 1B).

**Figure 2:**
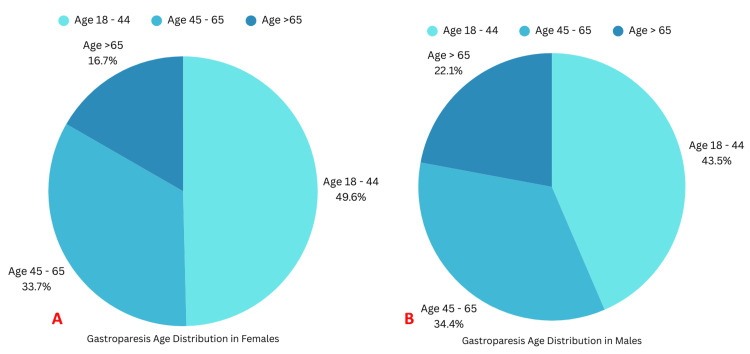
Age-wise distribution of females and males with gastroparesis A: Age-wise distribution of females with gastroparesis; B: Age-wise distribution of males with gastroparesis

Most patients were aged between 18 and 44 years (Figures 2A, 2B).

**Figure 3:**
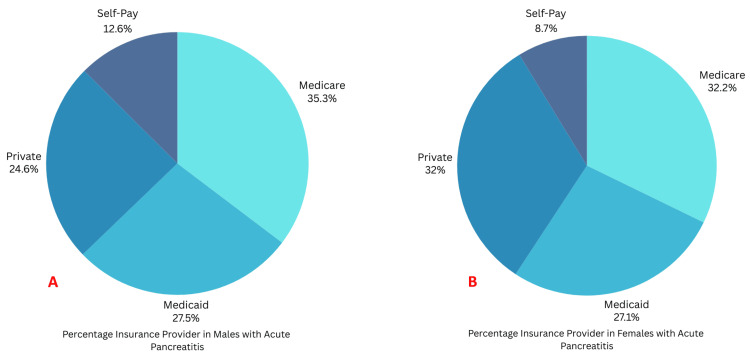
Distribution of type of insurance between females and males with gastroparesis A: Percentage-wise distribution of insurance providers for males with gastroparesis; B: Percentage-wise distribution of insurance providers for females with gastroparesis

Medicare was the primary payer for both females and males with GP (Figure 3A, 3B).

**Figure 4:**
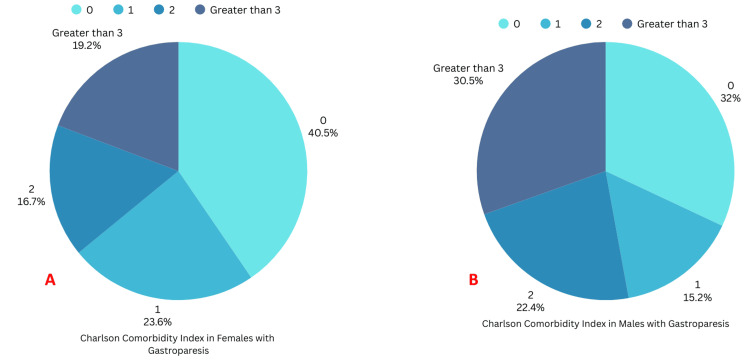
Charlson comorbidity index in females and males with gastroparesis A: Charlson Comorbidity Index in females with gastroparesis; B: Charlson Comorbidity Index in males with gastroparesis

Males had an overall higher CCI as compared to females (Figures 4A, 4B).

**Figure 5:**
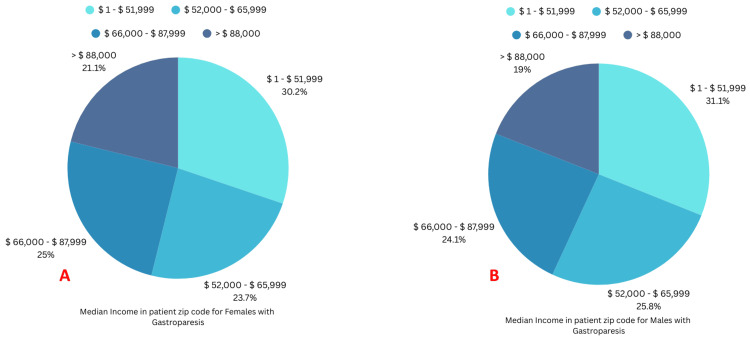
Median income distribution between females and males with gastroparesis A: Median income in patients' ZIP code for females with gastroparesis; B: Median income in patients' ZIP code for males with gastroparesis

The income of the majority of patients with GP was between $1 to $ 51,999 (Figures 5A, 5B).

**Figure 6:**
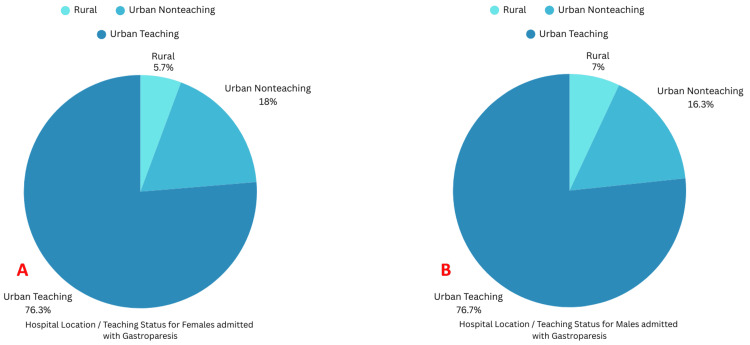
Teaching status of the hospitals where females and males with gastroparesis were admitted A: Location/teaching status of hospitals where females were admitted with gastroparesis; B: Location/teaching status of hospitals where males were admitted with gastroparesis

Most patients were admitted to urban teaching hospitals (Figures 6A, 6B).

**Figure 7:**
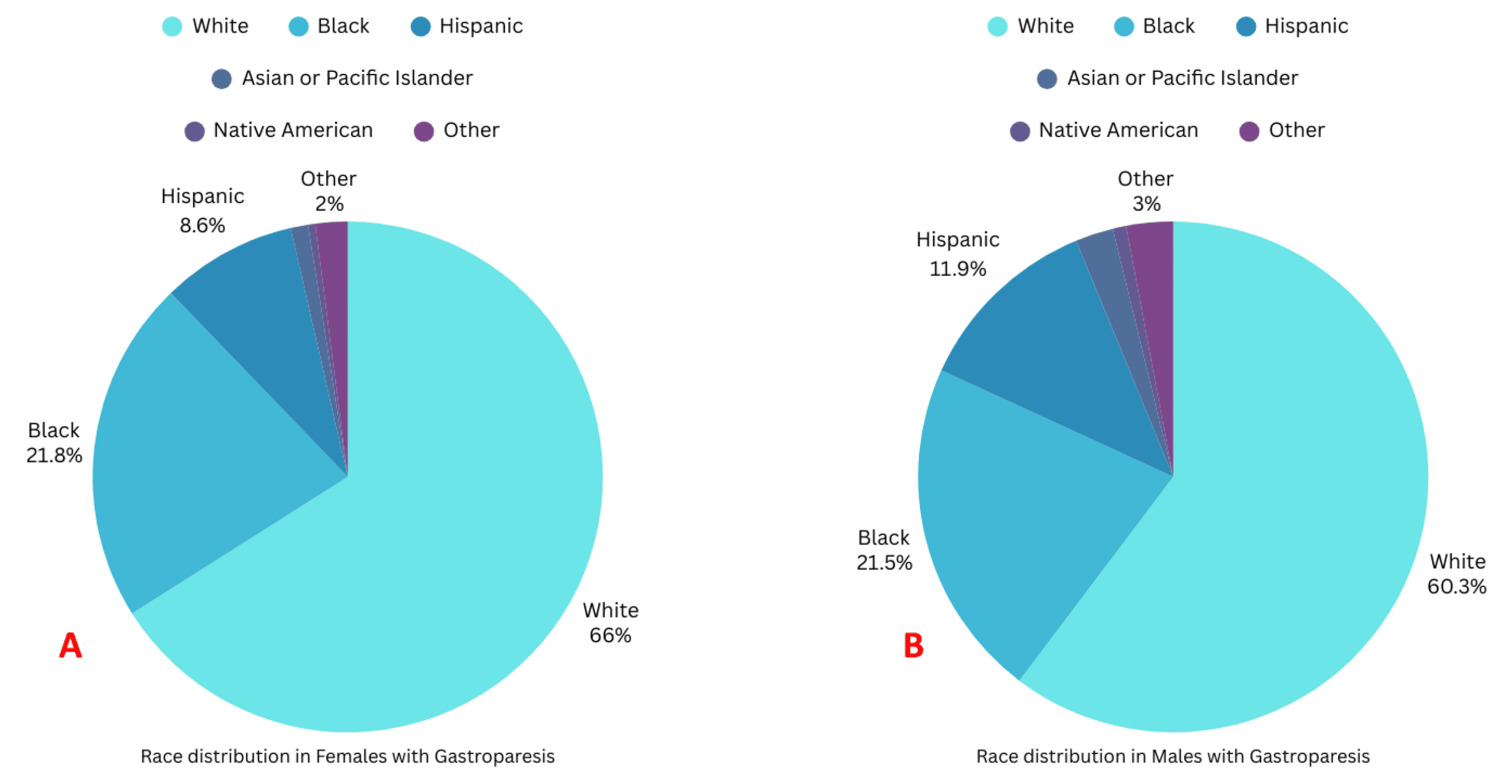
Race-based distribution of females and males admitted with gastroparesis A: Race-based distribution of females admitted with gastroparesis; B: Race-based distribution of males admitted with gastroparesis

The most prevalent race was Caucasian (Figures 7A, 7B).

**Figure 8:**
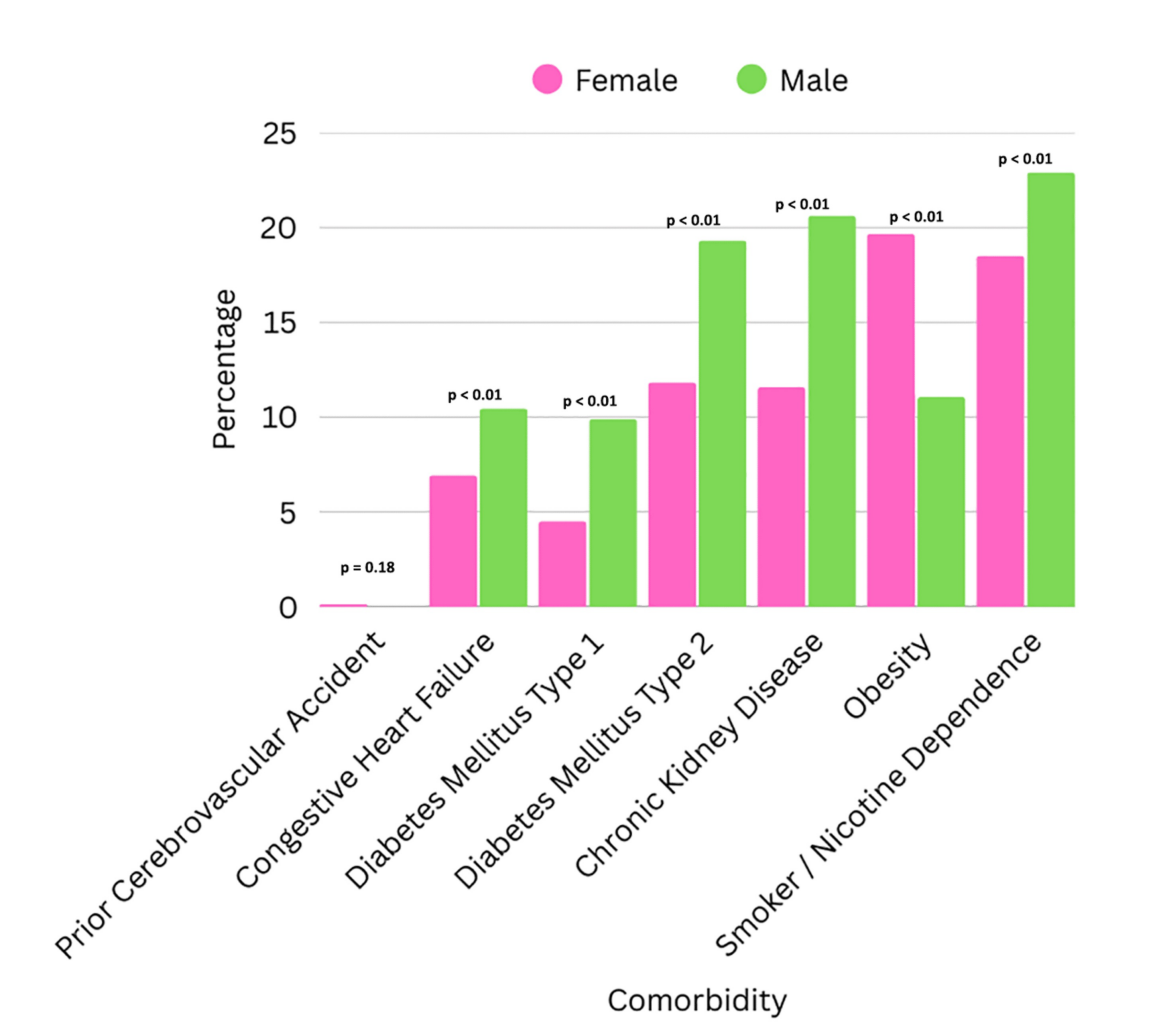
Comparison of comorbidities between females and males admitted with gastroparesis

We noticed that male patients with GP had a higher prevalence of comorbid conditions such as DM type 1 (DM1) (9.90% vs 4.52%, P <0.01), congestive heart failure (CHF) (10.45% vs 6.93%, P <0.01), chronic kidney disease (CKD) (20.62% vs 11.58%, P <0.01), DM type 2 (DM2) (19.31% vs 11.83%, P <0.01), and smoking/nicotine dependence (22.91% vs 18.51%, P <0.01). We noted female patients had a higher prevalence of obesity (19.66% vs 11.07%, P <0.01) and, although not statistically significant, prior cerebrovascular accident (CVA) (0.13% vs 0.00%, P=0.18) (Figure 8).

Patient and hospital characteristics of males and females with GP are presented in Table 1.

**Table 1:** Patient and hospital characteristics of male and female patients admitted with gastroparesis A p-value of less than 0.05 was considered statistically significant across all analyses. The Student t-test was used to calculate the p-value for the mean age and gender. The chi-square test was used to calculate the p-value for variables like insurance provider, Charlson Comorbidity Index, median income in patient zip code, hospital region, hospital location/teaching status, hospital size, race, prior cerebrovascular accident, congestive heart failure, diabetes mellitus type 1, diabetes mellitus type 2, chronic kidney disease, obesity, smoking/tobacco dependence, and gender.

Variable	Female	Male	P-value
Gender	23,886 (76.77%)	7,228 (23.23%)	
Mean age in years	47.2	47.2	< 0.01
Etiology			-
Diabetic	3,885 (64.80%)	2,110 (35.20%)	
Non-diabetic	20,002 (79.63%)	5,117 (20.37%)	
Age category (in years)			< 0.01
18-44	11,847 (49.60%)	3,146 (43.53%)	
45-65	8,052 (33.71%)	2,486 (34.39%)	
> 65	3,987 (16.69%)	1,596 (22.08%)	
Insurance provider			< 0.01
Medicare	9,053 (37.90%)	2,905 (40.19%)	
Medicaid	6,198 (25.95%)	1,898 (26.26%)	
Private	7,691 (32.20%)	1,914 (26.48%)	
Self-pay	944 (3.95%)	511 (7.07%)	
Charlson Comorbidity Index			< 0.01
0	9,662 (40.45%)	2,311 (31.97%)	
1	5,646 (23.64%)	1,096 (15.16%)	
2	3,982 (16.67%)	1,620 (22.42%)	
3 or more	4,596 (19.24%)	2,201 (30.45%)	
Median income in patients' zip code			0.19
$ 1 - $ 51,999	7,211 (30.19%)	2,246 (31.07%)	
$ 52,000 - $ 65,999	5,659 (23.69%)	1,866 (25.82%)	
$ 66,000 - $ 87,999	5,967 (24.98%)	1,740 (24.07%)	
> $ 88,000	5,049 (21.14%)	1,376 (19.04%)	
Hospital region			< 0.01
Northeast	4,290 (17.96%)	1,331 (18.41%)	
Midwest	4,242 (17.76%)	1,446 (20.00%)	
South	11,253 (47.11%)	3,021 (41.80%)	
West	4,101 (17.17%)	1,430 (19.79%)	
Hospital location/Teaching status			0.10
Rural	1,354 (5.67%)	505 (6.99%)	
Urban nonteaching	4,302 (18.01%)	1,180 (16.33%)	
Urban teaching	18,230 (76.32%)	5,543 (76.68%)	
Hospital size			< 0.01
Small	5,191 (21.73%)	1,536 (21.25%)	
Medium	6,437 (26.95%)	1,961 (27.13%)	
Large	12,258 (51.32%)	3,731 (51.62%)	
Race			< 0.01
White	15,772 (66.03%)	4,359 (60.31%)	
Black	5,200 (21.77%)	1,557 (21.54%)	
Hispanic	2,054 (8.60%)	863 (11.94%)	
Asian or Pacific Islander	265 (1.11%)	173 (2.40%)	
Native American	108 (0.45%)	62 (0.85%)	
Other	487 (2.04%)	214 (2.96%)	
Comorbidity			
Prior cerebrovascular accident	31 (0.13%)	0 (0.00%)	0.18
Congestive heart failure	1,655 (6.93%)	755 (10.45%)	< 0.01
Diabetes mellitus type 1	1,080 (4.52%)	715 (9.9%)	< 0.01
Diabetes mellitus type 2	2,826 (11.83%)	1,396 (19.31%)	< 0.01
Chronic kidney disease	2,766 (11.58%)	1,490 (20.62%)	< 0.01
Obesity	4,696 (19.66%)	800 (11.07%)	< 0.01
Smoker/Nicotine dependence	4,421 (18.51%)	1,656 (22.91%)	< 0.01

Length of hospital stay

We used linear regression analysis to calculate the mean length of hospital stay in males versus females with GP. We adjusted for possible confounders, including the CCI, age, race, hospital teaching status, hospital bed size, patients’ insurance status, and hospital location, using multivariate linear regression analysis. We found that the mean LOS in all patients admitted with GP was 4.91 days. The mean LOS in males was 4.29 days and 5.11 days in females. We found that female patients with GP had longer hospital LOS than males (+0.88 days, 95% CI: 0.53-1.29, P <0.01) after adjusting for confounders using multivariate linear regression analysis. This difference was statistically significant.

Total hospitalization charges

We used linear regression analysis to calculate the mean total hospitalization charges in males versus females with GP. We adjusted for possible confounders, including the CCI, age, race, hospital teaching status, hospital bed size, patients’ insurance status, and hospital location, using multivariate linear regression analysis. We found that the mean total hospitalization charges for all patients admitted with GP were $50,615. The mean total hospitalization charges for males were $44,874, and for females were $52,371. Female patients with GP had higher total hospitalization charges as compared to males (+ $9,129.4, 95% CI: 4,946.0-13,312.7, P <0.01) after adjusting for confounders using multivariate linear regression analysis. This difference was statistically significant.

ARF

We used logistic regression analysis to calculate the odds of ARF in males versus females with GP. We adjusted for possible confounders, including the CCI, age, race, hospital teaching status, hospital bed size, patients’ insurance status, and hospital location, using multivariate logistic regression analysis. We found that the overall incidence of ARF in all patients admitted with GP was 4,555 (14.6%). 1,667 (23.06%) males with GP developed ARF, and 2,888 (12.09%) females with GP developed ARF. Females with GP had 52% lower odds of developing ARF as compared to males (adjusted odds ratio (aOR) 0.48, 95% CI: 0.41-0.56, P <0.01) after adjusting for confounders using multivariate logistic regression analysis. This difference was statistically significant.

VTE

We used logistic regression analysis to calculate the odds of VTE in males versus females with GP. We adjusted for possible confounders, including the CCI, age, race, hospital teaching status, hospital bed size, patients’ insurance status, and hospital location, using multivariate logistic regression analysis. We found that the overall incidence of VTE in patients with GP was 335 (1.0%) patients. Fifty (0.7%) males and 285 (1.2%) females with GP developed VTE. Females had a 69% higher likelihood of developing VTE as compared to males (aOR 1.69, 95% CI: 0.83-3.44, P=0.147) after adjusting for confounders using multivariate logistic regression analysis. This difference was, however, not statistically significant. We believe the p-value was not statistically significant because of the low number of patients with GP who had VTE. 

Sepsis

We used logistic regression analysis to calculate the odds of sepsis in males versus females with GP. We adjusted for possible confounders, including the CCI, age, race, hospital teaching status, hospital bed size, patients’ insurance status, and hospital location, using multivariate logistic regression analysis. We found that overall, 775 (2.5%) patients with GP developed sepsis. Two hundred and sixty (3.6%) males and 515 (2.16%) females with GP developed sepsis. Females had 40% lower odds of developing sepsis than males (aOR: 0.60, 95% CI: 0.43-0.85, P <0.01) after adjusting for confounders using multivariate logistic regression analysis. This difference was statistically significant.

Shock

We used logistic regression analysis to calculate the odds of shock in males versus females with GP. We adjusted for possible confounders, including the CCI, age, race, hospital teaching status, hospital bed size, patients’ insurance status, and hospital location, using multivariate logistic regression analysis. We found that overall, 125 (0.4%) patients with GP developed shock. Forty-five (0.6%) males and 80 (0.3%) females with GP developed shock. Females with GP had 46% lower odds of developing shock than males with GP (aOR: 0.54, 95% CI: 0.24-1.22, P=0.143) after adjusting for confounders using multivariate logistic regression analysis. This difference was, however, not statistically significant. We believe the p-value was not statistically significant because of the low number of patients with GP who had shock. 

ICU admission

We used logistic regression analysis to calculate the odds of ICU admission in males versus females with GP. We adjusted for possible confounders, including the CCI, age, race, hospital teaching status, hospital bed size, patients’ insurance status, and hospital location, using multivariate logistic regression analysis. We found that 1950 (6.3%) patients with GP required ICU admission. Five hundred and eighty-five (8.1%) males and 1,365 (5.7%) females with GP required ICU admission. Females with GP had a 27% lower likelihood of requiring ICU admission as compared to males with GP (aOR 0.73, 95% CI: 0.57-0.92, P <0.01) after adjusting for confounders using multivariate logistic regression analysis. This difference was statistically significant.

In-hospital mortality

We used logistic regression analysis to calculate the odds of in-hospital mortality in males versus females with GP. We adjusted for possible confounders, including the CCI, age, race, hospital teaching status, hospital bed size, patients’ insurance status, and hospital location, using multivariate logistic regression analysis. Overall, in-hospital mortality occurred in 75 (0.2%) patients with GP. Fifty (0.69%) males and 25 (0.1%) females with GP died as inpatients. Females had 85% lower odds of in-hospital mortality as compared to males (aOR: 0.15, 95% CI: 0.05-0.45, P <0.01) after adjusting for confounders using multivariate logistic regression analysis. This difference was statistically significant.

## Discussion

Females are more susceptible to developing GP as compared to males; hence, GP is more prevalent in females. The rationale behind this gender inequality remains unclear. Several hypotheses have been proposed, such as the decreased gastric emptying rate and the influence of elevated sex-steroid hormones in females [9]. We conducted this study to examine the gender-related differences in outcomes of patients hospitalized with GP. To the best of our knowledge, this is the most updated analysis on gender-related outcomes in patients hospitalized with GP. We used the NIS database to estimate GP-related hospitalizations from 2020 to 2022, which is representative of approximately 95% of the U.S. population.

We noticed that of the 31,114 patients with GP, 23,886 (76.77%) were females and 7,228 (23.23%) were males. Our results show that a large proportion of patients hospitalized for GP were females. Our results are consistent with prior epidemiologic studies that show that 70% to 90% of patients with GP are females, and they outnumber males by a 4:1 ratio [10-15]. Jung et al. reported in their population-based study involving 3,604 residents that the incidence of GP in women was four times higher as compared to men [11]. Hirsch et al. reported an increase of 147% in diabetic GP emergency department visits, and nearly 70% of these patients were females [16]. We believe that GP is more symptomatic in females compared to males, resulting in females requiring higher hospitalization rates [17]. In their case series of 243 patients, Parkman et al. reported that females reported more severe nausea, satiety, and overall GP symptoms as compared to males [10].

We noted that females had a longer LOS as compared to males with GP. There are multiple factors that would contribute to these findings. The increased severity of GP symptoms in females, as discussed previously, might require an extended stay. Estrogen, which is more prevalent in females, can slow down the gastric emptying process, which may worsen symptoms of GP. Progesterone also has a relaxant effect on smooth muscle, which can contribute to delayed gastric emptying. We believe that these hormonal effects may lead to more severe symptoms in women, requiring longer hospitalizations [18]. Psychological factors related to women may also contribute to longer LOS, such as women may be more likely to seek medical care for GP, which can lead to more frequent and longer hospital admissions for symptom management [19].

Our results showed that females had higher hospitalization charges for GP as compared to males, and we believe this was due to several contributing factors. As mentioned earlier, women with GP often have longer hospital stays than men. Longer stays directly increase the overall hospitalization charges, as the cost of room, medications, diagnostic tests, and healthcare services accumulates over time. Females with GP have more severe symptoms, which require more complex and specialized care. The need for additional medications and prolonged monitoring can result in higher treatment costs [10]. Female patients may undergo additional or more extensive diagnostic testing due to the complexity of their symptoms and also the higher prevalence of other autoimmune diseases; hence, they are likely to undergo autoimmune workups to rule out other conditions, all of which add to the cost of care [20].

We noted an increased incidence of ARF in males with GP as compared to females. The reason for this is not entirely understood, but several factors may contribute to this disparity. GP is often associated with diabetes, and males have a higher prevalence of diabetes than females. Diabetes increases the risk of diabetic nephropathy. Male patients also had a higher prevalence of CKD. Hence, we believe that due to the higher prevalence of diabetes and CKD in males as compared to females, male patients with GP had a higher risk of developing ARF than females when they experienced dehydration, nausea, and vomiting from GP exacerbation in the hospital.

We noted higher odds of sepsis in males than females. One of the reasons for this could be the higher prevalence of comorbid conditions in males, like DM, CKD, and CHF, than in females. Poor blood sugar control, as in diabetes, impairs immune function and increases susceptibility to infections, including sepsis [21]. Males also had a higher prevalence of CHF, which can predispose them to poor circulation and compromised immune responses [22]. Both of these factors can increase the risk of infections progressing to sepsis. Females generally have more severe symptoms of GP, and maybe males seek treatment for GP later than females. We believe that the delayed recognition or treatment of GP in males leads to more severe and prolonged disease and complications like dehydration, electrolyte imbalances, or even bowel ischemia, all of which can create a more favorable environment for infection, increasing the risk of sepsis [19]. There is also an innate difference between male and female immune systems. Females typically have stronger immune responses, which can provide better protection against infections. Males, on the other hand, may have a less robust immune response, making them more susceptible to infections like sepsis when complications arise from GP [23].

We noted that females with GP tend to have a higher risk of thromboembolism compared to males. However, the p-value was not statistically significant, likely because the population of GP patients with VTE was too small to achieve statistical significance. We believe several factors contribute to females having a higher risk of VTE than males. Females have higher levels of estrogen and progesterone hormones that increase the risk of clot formation and VTE in females [24]. We noted obesity to be more prevalent in GP females, which also increases the risk of thromboembolism. Obesity is linked to inflammation, increased clotting factors, and poor circulation, all of which contribute to clot formation [25]. Females with GP had longer or more frequent hospitalizations due to the severity of their symptoms. Hospital stays often involve prolonged immobility, which is a significant risk factor for the development of deep vein thrombosis (DVT) and pulmonary embolism (PE). The combination of extended immobility, dehydration, and the underlying hypercoagulable state increases the likelihood of thromboembolic events. 

Our results showed that males had a higher incidence of shock, ICU admission, and in-hospital mortality as compared to females. We believe, as previously discussed, the higher risk of sepsis, ARF, poor immune response, seeking late treatment, and higher prevalence of comorbidities were all responsible for the higher incidence of shock, ICU admission, and in-hospital mortality in males as compared to females. Women are more likely to seek medical attention for GP issues, leading to earlier diagnosis and treatment of GP compared to men, and hence have better overall outcomes [18, 26].

Our study is significant because it underscores the higher inpatient mortality and overall poorer outcomes of GP in males compared to females. Our study alerts physicians that although females are more commonly hospitalized with GP, males have higher mortality and morbidity associated with GP and require closer monitoring to prevent worse clinical outcomes. We believe early involvement of gastroenterology services for GP flares in males and nephrology services in case patients have ARF can prevent worse clinical outcomes and improve outcomes in males hospitalized with GP.

Our study has several key strengths. It draws on a large, nationally representative sample from the NIS database, which includes broad geographic coverage across the United States. This enhances the generalizability of our findings and increases the statistical power of the analysis while also minimizing the risk of selection bias. Unlike many epidemiologic studies that rely on data from a single state or a limited number of centers, our study offers a more expansive and representative perspective. Furthermore, we reinforced the robustness of our results by using multivariate regression techniques to control for numerous potential confounders, such as age, race, gender, income level, comorbidity burden (CCI), and various hospital-related factors.

Our study is subject to some limitations as well. It relies on the NIS database, which uses ICD-10 codes for diagnostic classification. This introduces the possibility of misclassification, as some patients may have been incorrectly coded as having GP, potentially biasing the results. We also included patients with a primary diagnosis of nausea and vomiting and a secondary diagnosis of GP in our patient population, assuming that the primary diagnosis of nausea and vomiting in these patients is likely a GP flare. This might have included some patients who had nausea and vomiting not secondary to GP flare but other causes. However, the large sample size likely reduces the overall impact of such inaccuracies. Due to the observational nature of the NIS and its lack of randomization, we used multivariate regression to adjust for various patient- and hospital-level confounders. As a retrospective study, our analysis can reveal associations but cannot establish causality. Moreover, the NIS does not include data on the severity of GP, limiting our ability to assess outcomes based on disease severity.

## Conclusions

In clinical practice as well as in our study, we find that GP is more prevalent in females, and females seek more hospital care for GP than males. Around 76% of admitted patients were females, and the length of hospital stay and total hospitalization charges were higher in females. But, we found that males had overall worse clinical outcomes, including sepsis, shock, ICU admission, ARF, and mortality. We believe that symptoms of GP are more severe in females, and females seek more care for GP than males. This leads to earlier presentation to hospitals for females with GP and early intervention that reduces morbidity and mortality in females as compared to males. The same reason is why we observed higher hospital LOS and total hospitalization charges for females. We believe that symptoms of GP are milder in males, and hence, males seek treatment for GP later than females. Males, hence, present with advanced disease that leads to increased morbidity and mortality. That is the reason we believe we observed a higher incidence of ARF, sepsis, shock, ICU admission, and mortality in males than in females. Our study shows that males have worse clinical outcomes, and providers should hence treat males with GP aggressively to prevent morbidity and mortality.
